# Supplementary material for: Interventions and Impact of Pharmacist-Delivered Services for People Infected with COVID-19: A Systematic Review
Source: Healthcare (Basel). 2022 Aug 26;10(9):1630. doi: 10.3390/healthcare10091630 (PMC9498522; doi:10.3390/healthcare10091630)
Supplement: Supplementary file 1 [file healthcare-10-01630-s001.zip › healthcare-1858255-supplementary.pdf]

Supplementary Data

Details of key words databases searches

A. PubMed

#1 "COVID-19" [Supplementary Concept] OR (2019 novel coronavirus disease) OR (COVID19) OR (COVID-19 pandemic) OR (SARS-CoV-2 infection) OR (COVID-19 virus disease) OR (2019 novel coronavirus infection) OR (2019-nCoV infection) OR (coronavirus disease 2019) OR (coronavirus disease-19) OR (2019-nCoV disease) OR (COVID-19 virus infection) OR (COVID-19 vaccination) OR (COVID-19 immunization) OR (COVID-19 hesitancy)

#2 "Pharmacists"[Mesh] OR (Pharmacist) OR (Clinical Pharmacists) OR (Clinical Pharmacist) OR (Pharmacist, Clinical) OR (Pharmacists, Clinical) OR (Community Pharmacists) OR (Community Pharmacist) OR (Pharmacist, Community) OR (Pharmacists, Community) OR (Hospital Pharmacists) OR "Pharmacies"[Mesh] OR (Community Pharmacy) OR (Community Pharmacies) OR "Pharmacy Service, Hospital"[Mesh] OR (Pharmaceutical Service, Hospital) OR (Service, Hospital Pharmaceutical) OR (Hospital Pharmacy Services) OR (Pharmacy Services, Hospital) OR (Services, Hospital Pharmacy) OR (Service, Hospital Pharmaceutic) OR (Hospital Pharmacy Service) OR (Hospital Pharmaceutic Service) OR (Hospital Pharmaceutic Services) OR (Pharmaceutic Services, Hospital) OR (Services, Hospital Pharmaceutic) OR (Pharmaceutic Service, Hospital) OR (Hospital Pharmaceutical Service) OR (Hospital Pharmaceutical Services) OR (Pharmaceutical Services, Hospital) OR (Services, Hospital Pharmaceutical) OR (Service, Hospital Pharmacy) OR (Pharmacy Service, Clinical) OR (Service, Clinical Pharmacy) OR (Clinical Pharmacy Services) OR (Pharmacy Services, Clinical) OR (Services, Clinical Pharmacy) OR (Clinical Pharmacy Service) OR "Pharmaceutical Services"[Mesh] OR (Services, Pharmaceutic) OR (Services, Pharmacy) OR (Pharmaceutic Services) OR (Pharmaceutic Service) OR (Service, Pharmaceutic) OR (Services, Pharmaceutical) OR (Pharmaceutical Service) OR (Service, Pharmaceutical) OR (Pharmacy Services) OR (Pharmacy Service) OR (Service, Pharmacy) OR (Pharmaceutical Care) OR (Care, Pharmaceutical) OR "Medication Therapy Management"[Mesh] OR (Management, Medication Therapy) OR (Therapy Management, Medication) OR (Drug Therapy Management) OR (Management, Drug Therapy) OR (Therapy Management, Drug)

#1 AND #2

Filter: publication date from 2019/12/01.

B. Embase

#1 'covid 19'/exp OR 'covid 19' OR 'sars coronavirus'/exp OR 'sars cov 2' OR 'coronavirus disease 2019'/exp OR coronavirus OR '2019 ncov infection' OR '2019 ncov disease'

#2 'pharmacist'/exp OR pharmacist OR pharmacists OR 'community pharmacist'/exp OR 'community pharmacists' OR 'clinical pharmacist'/exp OR 'clinical pharmacists' OR 'hospital pharmacist'/exp OR 'hospital pharmacists' OR 'hospital pharmacy'/exp OR 'hospital pharmacy' OR 'clinical pharmacy'/exp OR 'clinical pharmacy' OR 'pharmacy (shop)'/exp OR 'community pharmacy' OR pharmacy OR pharmacies OR 'pharmaceutical care'/exp OR 'pharmaceutical care' OR 'medication therapy management'/exp OR 'medication therapy management' OR 'drug therapy management' OR 'medication management'

#1 AND #2

Filter: publication date from 2019/12/01.

C. Scopus

#1 TITLE-ABS-KEY (covid 19 OR coronavirus)

#2 TITLE-ABS-KEY (pharmacist OR pharmacists OR (community AND pharmacists) OR (clinical AND pharmacists) OR (hospital AND pharmacists) OR (hospital AND pharmacy) OR (clinical AND pharmacy) OR (community AND pharmacy) OR pharmacy OR pharmacies OR (pharmaceutical AND care) OR (medication AND therapy AND management) OR (drug AND therapy AND management) OR (medication AND management))

#1 AND #2

Filter: publication date from 2019/12/01.

Table S1: PRISMA Checklist

Section and Topic	Item #	Checklist item	Location where item is reported
**TITLE**			
Title	1	Identify the report as a systematic review.	Title
**ABSTRACT**			
Abstract	2	See the PRISMA 2020 for Abstracts checklist.	Abstract
**INTRODUCTION**			
Rationale	3	Describe the rationale for the review in the context of existing knowledge.	Introduction 3rd paragraph
Objectives	4	Provide an explicit statement of the objective(s) or question(s) the review addresses.	Introduction last paragraph
**METHODS**			
Eligibility criteria	5	Specify the inclusion and exclusion criteria for the review and how studies were grouped for the syntheses.	Methods subsection: Study Selection Criteria
Information sources	6	Specify all databases, registers, websites, organisations, reference lists and other sources searched or consulted to identify studies. Specify the date when each source was last searched or consulted.	Methods subsection: Information Sources and Databases Search Strategy
Search strategy	7	Present the full search strategies for all databases, registers and websites, including any filters and limits used.	Methods subsection: Information Sources and Databases Search Strategy and Supplementary file tables
Selection process	8	Specify the methods used to decide whether a study met the inclusion criteria of the review, including how many reviewers screened each record and each report retrieved, whether they worked independently, and if applicable, details of automation tools used in the process.	Methods subsection: Data Screening and Extraction
Data collection process	9	Specify the methods used to collect data from reports, including how many reviewers collected data from each report, whether they worked independently, any processes for obtaining or confirming data from study investigators, and if applicable, details of automation tools used in the process.	Methods subsection: Data Screening and Extraction
Data items	10a	List and define all outcomes for which data were sought. Specify whether all results that were compatible with each outcome domain in each study were sought (e.g. for all measures, time points, analyses), and if not, the methods used to decide which results to collect.	Methods subsection: Data Screening and Extraction
	10b	List and define all other variables for which data were sought (e.g. participant and intervention characteristics, funding sources). Describe any assumptions made about any missing or unclear information.	Methods subsection: Data Screening and Extraction
Study risk of bias assessment	11	Specify the methods used to assess risk of bias in the included studies, including details of the tool(s) used, how many reviewers assessed each study and whether they worked independently, and if applicable, details of automation tools used in the process.	Methods subsection: Risk of Bias Assessment
Effect measures	12	Specify for each outcome the effect measure(s) (e.g. risk ratio, mean difference) used in the synthesis or presentation of results.	Methods subsection: Data Synthesis
Synthesis methods	13a	Describe the processes used to decide which studies were eligible for each synthesis (e.g. tabulating the study intervention characteristics and comparing against the planned groups for each synthesis (item #5)).	Methods subsection: Data Synthesis

Section and Topic	Item #	Checklist item	Location where item is reported
	13b	Describe any methods required to prepare the data for presentation or synthesis, such as handling of missing summary statistics, or data conversions.	Methods subsection: Data Synthesis
	13c	Describe any methods used to tabulate or visually display results of individual studies and syntheses.	Methods subsection: Data Synthesis
	13d	Describe any methods used to synthesize results and provide a rationale for the choice(s). If meta-analysis was performed, describe the model(s), method(s) to identify the presence and extent of statistical heterogeneity, and software package(s) used.	Methods subsection: Data Synthesis
	13e	Describe any methods used to explore possible causes of heterogeneity among study results (e.g. subgroup analysis, meta-regression).	Methods subsection: Data Synthesis
	13f	Describe any sensitivity analyses conducted to assess robustness of the synthesized results.	Methods subsection: Data Synthesis
Reporting bias assessment	14	Describe any methods used to assess risk of bias due to missing results in a synthesis (arising from reporting biases).	Methods subsection: Risk of Bias Assessment
Certainty assessment	15	Describe any methods used to assess certainty (or confidence) in the body of evidence for an outcome.	NR
**RESULTS**			
Study selection	16a	Describe the results of the search and selection process, from the number of records identified in the search to the number of studies included in the review, ideally using a flow diagram.	Results first paragraph and Figure 1
	16b	Cite studies that might appear to meet the inclusion criteria, but which were excluded, and explain why they were excluded.	Results first paragraph and Figure 1
Study characteristics	17	Cite each included study and present its characteristics.	Results subsection: Characteristics of the Included Studies
Risk of bias in studies	18	Present assessments of risk of bias for each included study.	Results subsection: Risk of Bias
Results of individual studies	19	For all outcomes, present, for each study: (a) summary statistics for each group (where appropriate) and (b) an effect estimate and its precision (e.g. confidence/credible interval), ideally using structured tables or plots.	Results subsection: Outcomes of Pharmacist Interventions, Tables 1 & 3
Results of syntheses	20a	For each synthesis, briefly summarise the characteristics and risk of bias among contributing studies.	Results subsection: Risk of Bias & Characteristics of the Included Studies
	20b	Present results of all statistical syntheses conducted. If meta-analysis was done, present for each the summary estimate and its precision (e.g. confidence/credible interval) and measures of statistical heterogeneity. If comparing groups, describe the direction of the effect.	Results subsection: Outcomes of Pharmacist Interventions
	20c	Present results of all investigations of possible causes of heterogeneity among study results.	Results subsection: Outcomes of

Section and Topic	Item #	Checklist item	Location where item is reported
			Pharmacist Interventions
	20d	Present results of all sensitivity analyses conducted to assess the robustness of the synthesized results.	NR
Reporting biases	21	Present assessments of risk of bias due to missing results (arising from reporting biases) for each synthesis assessed.	Risk of bias Table 1 & Supplementary file table 4.
Certainty of evidence	22	Present assessments of certainty (or confidence) in the body of evidence for each outcome assessed.	Certainty of evidence was not assessed.
**DISCUSSION**			
Discussion	23a	Provide a general interpretation of the results in the context of other evidence.	Discussion: Paragraph 1-3
	23b	Discuss any limitations of the evidence included in the review.	Discussion subsection: Strengths and weaknesses
	23c	Discuss any limitations of the review processes used.	Discussion subsection: Strengths and weaknesses
	23d	Discuss implications of the results for practice, policy, and future research.	Discussion: Paragraph 3-4
**OTHER INFORMATION**			
Registration and protocol	24a	Provide registration information for the review, including register name and registration number, or state that the review was not registered.	Abstract & Methods
	24b	Indicate where the review protocol can be accessed, or state that a protocol was not prepared.	Accessible
	24c	Describe and explain any amendments to information provided at registration or in the protocol.	No amendments done
Support	25	Describe sources of financial or non-financial support for the review, and the role of the funders or sponsors in the review.	Title page
Competing interests	26	Declare any competing interests of review authors.	Title page
Availability of data, code and other materials	27	Report which of the following are publicly available and where they can be found: template data collection forms; data extracted from included studies; data used for all analyses; analytic code; any other materials used in the review.	Within manuscript, tables and supplementary files

Table S2: PICOS Inclusion criteria

**PICOS Terms**	
**Population**	People infected with COVID-19
**Intervention**	Any pharmacist intervention (Pharmaceutical care, education, etc.) contributes to managing the medication-related problems in COVID-19 patients.

**Comparator**	Usual care for comparative studies with people not infected with COVID-19 and no comparator for cross-sectional studies.
**Outcomes**	Identification of medication errors, correction of drug-related problems, optimization of therapy, the physician acceptance rate of interventions
**Study designs**	Randomized trials, non-randomized studies, cross-sectional, retrospective or prospective cohort, or descriptive studies

Table S3. Detailed quality assessment of studies by Newcastle-Ottawa Scale of each included study.

Score interpretation: *7-9 high quality, 4-6 high risk, and 0-3 very high risk of bias.

**Study**	**Selection**				**Comparability**	**Outcome**			**Total score***
	Representativeness of exposed cohort	Selection of non-exposed cohort	Ascertainment of exposure	Demonstration that outcome of interest was not present at the start of study	Comparability of cohorts on the basis of the design or analysis	Assessment of outcome	Was follow-up long enough for outcomes to occur	Adequacy of follow up of cohorts	
Perez et al. 2020	*	*	*	-	*	*	*	**	8
Ibrahim et al. 2020	*	*	*	-	*	*	*	*	7
Surapat et al. 2020	*	-	*	-	-	*	-	-	3
Collins et al. 2020	*	-	*	-	-	*	*	*	5
Alwhaibi et al. 2021	*	-	*	-	-	*	*	*	5
Wang et al. 2021	*	-	*	-	-	*	-	-	3
Al-Quteimat at al. 2022	*	-	*	-	-	*	*	*	5
